# The Evolution of Live Patient Viewing in the Era of COVID-19: Survey Study

**DOI:** 10.2196/39952

**Published:** 2022-10-18

**Authors:** Rachel Sally, Katharina Shaw, Roger Ho

**Affiliations:** 1 Ronald O Perelman Department of Dermatology New York University Grossman School of Medicine New York, NY United States

**Keywords:** COVID-19 pandemic, live patient viewing, dermatology education, dermatology, COVID-19, residency program, residency, dermatology residency, medical education, virtual education, didactic

Over two years into the COVID-19 pandemic, the effects of this unprecedented crisis continue to unfold. Despite increasing vaccination rates and relaxation of “social distancing,” avoiding extraneous person-to-person contact remains the gold standard, particularly in health care settings. In dermatology, where close examination of the skin is paramount, this policy has far-reaching consequences. Dermatology resident education has been particularly disrupted because live patient viewing sessions (LPVSs)—a longstanding pillar of dermatology training—have been infeasible when “distancing” is required. While much of dermatology education will likely return to baseline post pandemic, the fate of LPVSs remains unclear. We thus aimed to assess the baseline integration of LPVSs, identify pandemic modifications, and ascertain permanent pedagogical changes.

In September 2020, an institutional review board–approved web-based survey was sent to 123 US dermatology residency programs (Multimedia Appendix 1). The survey queried demographics and curricular integration of LPVSs before, during, and after the pandemic. Of 123 contacted, 44 (35.8%) surveys were completed. Most programs hosted LPVSs prepandemic (n=39, 89%), and the majority supplemented these live sessions with virtual cases (n=35, 80%; Table 1). All programs canceled LPVSs at the onset of the pandemic, with most substituting virtual cases (n=40, 91%). Regarding LPVS resumption post pandemic, 13 (30%) reported they would restart, 25 (57%) were ambivalent at the time, and 6 (14%) reported LPVSs would not recommence. Fisher exact test revealed no significant relationship between geography and LPVS resumption (*P*=.21). Of the programs reporting that LPVSs would *not* resume, the majority believed virtual cases were sufficient to replace live sessions. All programs committed to restarting LPVSs will continue incorporating virtual cases.

The 19th-century physician Osler [1] revolutionized medical education with his emphasis on bedside training. His tradition of live patient viewing has been maintained by academic dermatology departments nationwide, as reflected by our results: prior to the COVID-19 pandemic, 39 (89%) dermatology programs hosted LPVSs, with the majority (n=40, 90%) during grand rounds, and over half (51%) hosting LPVSs several times a month. LPVSs are consistently ranked highly among residents, and our results suggest similar sentiments among program leadership, with 34 (77%) viewing LPVSs as integral to resident education and 36 (82%) believing LPVSs facilitate collaboration (Table 2) [2-5].

Yet despite the value of LPVSs to trainees and faculty alike, our results demonstrate a surfeit of uncertainty in reintroducing in-person sessions, with 25 (57%) respondents unsure about preserving LPVSs. At least 6 surveyed programs discontinued LPVSs altogether. Whether additional programs ultimately decide against readopting LPVSs remains uncertain. Our results suggest an overwhelming trend toward incorporating virtual patient conferences into didactic curricula. As vaccination rates increase and the COVID-19 pandemic wanes, the proportional fates of live and virtual patient viewing sessions within dermatology will doubtlessly declare themselves. As Osler [1] wrote, “to study...the disease without books is to sail an uncharted sea, while to study books without patients is not to go to sea at all.”

**Table 1:** Demographic and curricular integration results (N=44).

Participant responses			Participants, n (%)
**Demographic characteristics**			
	**Geographic location**		
		Northeast	10 (23)
		Southeast	9 (20)
		Midwest	13 (30)
		Northwest	4 (9)
		Southwest	8 (18)
	**Program size (residents)**		
		≤8	8 (18)
		9-18	27 (61)
		≥19	9 (20)
**Curricular integration**			
	**Live PVS^a^**		
		Pre–COVID-19^b^	39 (89)
		During COVID-19^c^	0 (0)
		Anticipated return post COVID-19^d^	13 (30)
	**Virtual PVS**		
		Pre–COVID-19^b^	35 (80)
		During COVID-19^c^	40 (91)
		Anticipated return post COVID-19^d^	17 (39)

^a^PVS: patient viewing session.

^b^Pre–COVID-19 corresponds to prior to March 2020.

^c^Defined as March 2020 to time of the survey distribution (September 2020).

^d^A total of 25 participants were unsure at the time whether they would return to PVSs.

**Table 2:** Live PVS needs assessment survey results (N=44).

	Strongly agree, n (%)	Agree, n (%)	Neutral, n (%)	Disagree, n (%)
Integral part of resident education	21 (47)^a^	13 (30)^a^	4 (9)	1 (2)
Teach trainees morphology, differential diagnoses, and disease management	24 (55)	13 (30)	2 (5)	0 (0)
Provide opportunities for clinicopathological correlation	23 (52)	14 (32)	2 (5)	0 (0)
Useful for providing high-quality patient care	20 (45)	14 (32)	5 (11)	0 (0)
Useful for seeking other dermatologists’ opinions about diagnosis or management of difficult cases	26 (59)^b^	10 (23)^b^	3 (7)	0 (0)
Conducted in a humanistic manner	21 (48)	14 (32)	2 (5)	2 (5)
Has a set of rules/conduct guidelines that are consistently followed	17 (39)	16 (36)	3 (7)	3 (7)
Patients generally feel comfortable with being seen by a group of physicians	11 (25)	19 (43)	7 (16)	2 (5)
Patients view their participation in PVSs^c^ as worthwhile	15 (34)	19 (43)	4 (9)	1 (2)
Strengthen the physician-patient relationship	8 (18)	16 (36)	13 (30)	2 (5)

^a^A total of 77% (n=34) of respondents agree or strongly agree that live patient viewing sessions are an integral part of resident education.

^b^A total of 82% (n=36) of respondents agree or strongly agree that live patient viewing sessions help foster collaboration between physicians.

^c^PVS: patient viewing session.

## Appendix

Multimedia Appendix 1 Institutional review board–approved web-based survey via RedCap.

## Abbreviations

LPVS: live patient viewing session
