# Supplementary material for: The Evolution of Live Patient Viewing in the Era of COVID-19: Survey Study
Source: JMIR Dermatol. 2022 Oct 18;5(4):e39952. doi: 10.2196/39952 (PMC10012180; doi:10.2196/39952)
Supplement: Multimedia Appendix 1 [file derma_v5i4e39952_app1.docx]

**Online-only Supplement**

**Title:** The Evolution of Live Patient Viewing in the Era of COVID-19: Survey Study

**Contents:**

1. eAppendix

1. eAppendix: IRB-approved, web-based survey via RedCap.
